# Low-Flammability Hybrid Polymer Materials Based on Epoxy Oligomers and In Situ-Synthesized Zinc-Containing Microparticles

**DOI:** 10.3390/polym17243291

**Published:** 2025-12-11

**Authors:** Sergey Vladimirovich Borisov, Boris Andreevich Buravov, Daria Andreevna Kudryavtseva, Valentin Olegovich Kharlamov, Artem Aleksandrovich Kobelev, Stanislav Albertovich Trubachev, Marat Abdurakhmanovich Vaniev, Ivan Aleksandrovich Novakov

**Affiliations:** 1 Faculty of Chemical Technology, Volgograd Technical State University, 400005 Volgograd, Russia; byravov@ya.ru (B.A.B.); daschakud@yandex.ru (D.A.K.); harlamov_vo@mail.ru (V.O.K.); m.a.vaniev@mail.ru (M.A.V.); ianovakov@vstu.ru (I.A.N.); 2 Educational and Research Centre “Fire Safety of Civil and Industrial Buildings” of Academy of State Fire Service, Academy of the State Fire Service of the Ministry of Emergency Situations of Russia, 129366 Moscow, Russia; artemkobelev@yandex.ru; 3 Voevodsky Institute of Chemical Kinetics and Combustion, Siberian Branch of the Russian Academy of Sciences, 630090 Novosibirsk, Russia; trubachev@kinetics.nsc.ru

**Keywords:** DGEBA, microparticles, in situ, low flammability, zinc sulfate, phosphoric acid

## Abstract

This study addresses the drawbacks of traditional dispersed fire retardants—such as anisotropy, reduced strength, and poor filler impregnability—by developing in situ-formed hybrid epoxy composites. The materials, based on diglycidyl ether of bisphenol A and triethylenetetramine, were modified with a solution of zinc sulfate heptahydrate in orthophosphoric acid. This approach yielded near-spherical microparticles (6–16 µm) within the polymer matrix. The scientific novelty lies in investigating how such in situ particle formation affects material properties. The modification significantly enhanced fire resistance: char residue increased 1.7–2.2-fold, while total heat release, peak heat release rate, and smoke release were reduced by up to 60.5%, 40.2%, and 70%, respectively. The observed increase in the mass loss rate suggests that accelerated thermal-oxidative degradation promotes char formation. These findings, supported by scanning electron microscopy, energy-dispersive X-ray spectroscopy, and Fourier-transform infrared spectroscopy data, demonstrate the efficacy of the in situ strategy for creating high-performance, fire-safe epoxy composites.

## 1. Introduction

The modern industrial setting, involving a variety of critical sectors such as construction, transport, electronics, and aviation, has seen a dramatic expansion in the use of polymer matrix composites (PMCs) based on epoxy resins. Due to the possibility of directed formulation design, polymer composites enable the manufacturing of products with predetermined performance characteristics and a relatively low specific gravity, gaining an advantage over many metals [1,2,3]. However, most PMC products are characterized by high flammability, thus being in conflict with fire safety requirements [4,5]. This explains the need for seeking new, and, in particular, composite, materials.

A promising new area of research aiming to accomplish enhanced operational characteristics is the development of hybrid polymer materials [6,7]; researchers are combining the properties of metals and plastics, which significantly expands the functional capabilities of polymer matrix composites by improving their flame resistance, durability, and environmental stability. Powder-based fire retardants using magnesium and aluminum hydroxides are currently widely used. However, to provide effective action, they must be introduced in large quantities—up to 100% of the polymer by weight [8]. Despite having a positive effect on PMC fire resistance, dispersed additives as part of an epoxy composition tend to cause anisotropy and impair physical and mechanical characteristics and prevent efficient impregnation of reinforcing fillers due to a high hydrodynamic resistance. This limits the ability of epoxy resins with dispersed fillers to be used as binders. Furthermore, mixing requires the use of additional equipment with ambient condition control (temperature, mixing time, and rate) to achieve uniform distribution of particles in the matrix, which makes the process costly and labor-intensive. Therefore, the focus has increasingly been on fire retardants making use of phosphorus compounds [9,10,11] and metal nanoparticles [12,13,14]. The latter allow for the production of composites with enhanced adhesive and thermal performance [15,16]. In particular, epoxy composites based on a Zn-imidazole metal–organic framework and graphene oxide are known for corrosion protection. The study demonstrated reliable long-term anti-corrosion performance of such nanocomposite coatings when applied to a steel substrate for protecting metal surfaces from corrosion [17]. At the same time, the anisotropy problem resulting from additive deposition has not been completely resolved and was merely mitigated by a low content thereof and a small particle size.

One of the methods for creating nanocomposites with metallic nanoparticles is in situ reduction in nonpolar media at moderate temperatures using gold compounds, silver, and copper carboxylates as precursors [18,19,20,21,22,23,24]. This method of introducing metal-containing structures into epoxy composites to improve their fire resistance is of special interest. In particular, this can be accomplished by coupling epoxy oligomers with derivatives of phosphoric acid. The latter actively reacts with epoxy groups and proves extremely difficult to use separately as a modifying additive. Nevertheless, phosphoric acid appears quite promising as a curing and fire-retardant agent for epoxy resins [25,26,27,28,29,30]. Yet, in this case, it cannot be used as pure phosphoric acid and will require additives to inhibit interaction between phosphoric acid and epoxy groups [31,32,33]. We propose the use of inorganic zinc salts dissolved in H_3_PO_4_ as such additives. The application of these additives for the modification of epoxy-based polymers and the investigation of their effect on the properties of the resulting materials constitute the scientific novelty of this research.

The purpose of this paper is to study the effects of modifying epoxy oligomers with in situ-synthesized zinc-containing microparticles on the flammability levels as well as the physical and mechanical properties of cured polymer materials.

## 2. Materials and Methods

The research was based on the use of bisphenol A diglycidyl ether (DGEBA) (20 wt% epoxy groups, Poliko Retail LLC, Moscow, Russia) with triethylenetetramine (TETA) as a curing agent (97.2 wt%, Delamine BV, Meppel, The Netherlands). The TETA concentration, calculated to account for the equimolar ratio between amine and epoxy groups, was 10 parts per 100 parts of DGEBA.

The modifying additive was a solution obtained by combining zinc sulfate heptahydrate (99.5 wt%, Ruskhim LLC, Moscow, Russia) with orthophosphoric acid (73 wt%, Ruskhim LLC, Moscow, Russia) at 20–25 °C and maintaining the mixture at this temperature for 24 h. The concentration of orthophosphoric acid varied from 0.11 to 2.98 wt%, and that of zinc salt ranged from 7.5 × 10^−3^ to 189 × 10^−3^ wt%.

The component blending ratios are provided in Table 1.

It was found that the introduction of modifiers into a DGEBA + TETA mixture resulted in the formation of non-uniformities and lumps, critically impairing the process efficiency. This effect, however, is not observed when the modifier is first combined with DGEBA and, after a thorough mixing, the hardener is added to the mixture—resulting in a uniform, non-transparent white binder.

The epoxy compositions were cured at a temperature of 20–25 °C for 24 h, with subsequent thermal conditioning for 4 h at 80 °C.

The strength characteristics of the epoxy polymers were assessed by performing a three-point bending test according to the [34] procedure (method A) using a ZwickRoell Zwick Z 5.0 TH (ZwickRoell GmbH & Co. KG, Ulm, Germany) testing machine with a crosshead speed of 2 mm/min.

The effects of the modifier on the structure of the epoxy polymers and the curing process were studied according to the [35] procedure using a SIMEX FT-801 (SIMEX, Saint Petersburg, Russia) Fourier-transform infrared spectrometer with an attenuated total reflection (ATR) accessory equipped with a zinc selenide optical crystal.

The surface morphology (fractographic analysis) and elemental composition of the matrix and particles were studied using a Versa 3D DualBeam FEI dual-beam scanning electron microscope (Thermo Fisher Scientific, Hillsboro, OR, USA) fitted with an AZtecLive Expert (v6.1, Oxford Instruments NanoAnalysis, High Wycombe, UK) energy-dispersive analysis system and equipped with an Ultim Max 65 detector (Bayersikring AS, Bærum, Norway). The resulting microphotographs were processed in the FastStone Image Viewer 7.9 application (FastStone Soft, Toronto, ON, Canada). The key particle-size distribution metrics were calculated using the methodology described in reference [36].

The limiting oxygen indices of the developed polymers were determined according to the [37] procedure using an Oxygen Index Module (Concept Equipment Ltd., South Wonston, UK).

In response to the request for clarification, the experimental section has been revised to include the following critical details. The samples for cone calorimeter testing were square plates measuring 100 × 100 mm with a thickness of 4 mm. Prior to testing, all samples were conditioned according to the [38] procedure at a temperature of 23 ± 2 °C for 48 h. To eliminate edge effects and ensure uniform heating, the samples were wrapped on the bottom and sides with aluminum foil (thickness 0.05 mm) and mounted in the horizontal sample holder, exposing only the top surface to the radiant heat flux. The experiments were conducted on a Skyline SL-FL01 cone calorimeter (Skyline Instrument Ltd., Beijing, China) under a heat flux of 50 kW/m^2^, calibrated using a water-cooled radiometric flux gauge prior to the tests, in accordance with ISO 5660. The heat release rate was determined by oxygen consumption using a Siemens Oxymat paramagnetic oxygen analyzer (Siemens AG, Munich, Germany), which was calibrated with technical air (20.95% O_2_) and pure nitrogen (99.99% N_2_), following the standard procedure outlined in ISO 5660-1. Finally, the samples were weighed using a built-in analytical balance with a manufacturer’s stated accuracy of ±0.1 g. These clarifications have been incorporated into the revised manuscript.

The thermal-oxidative degradation process was studied according to the [39] procedure using a METTLER TOLEDO TGA/DSC 1 simultaneous thermal analysis device (Mettler-Toledo GmbH, Greifensee, Switzerland). The heating rate was 20 °C/min; the reaction gas velocity was 50 mL/min; the temperature range was 25–500 °C. The testing medium was air. The average sample weight was 13 mg (in powder form).

The water absorption levels of the epoxy polymers were determined according to the [40] procedure by holding them in distilled water at a temperature of 23–30 °C and thereafter measuring the pH of the aqueous medium using an Eco Titrator (Metrohm AG, Herisau, Switzerland) instrument with an LL-Solvotrode easyClean combined pH electrode (Metrohm AG, Herisau, Switzerland). To identify the ability of the modifying additive to leach out from epoxy polymers, the medium was studied after immersion by means of X-ray-fluorescence spectrometry on a Shimadzu EDX-8000 energy-dispersive spectrometer (Shimadzu Corporation, Kyoto, Japan). The quantitative analysis was carried out via the fundamental parameter method using the integrated software package.

The gel fraction content was determined by extraction carried out for 24 h in a Soxhlet apparatus using toluene (chemically pure, 99.8 wt%, AO Ekos-1, Moscow, Russia) as the solvent, with reference to the methodology described in [41].

The rheokinetics of curing a synthesized binder were studied according to the methodology described in [42] using a Brookfield LVDV-II + Pro programmable rotational viscometer (Brookfield Engineering Laboratories, Middleboro, MA, USA). The test was conducted in a stainless-steel HT-2 measuring chamber at a temperature of 23 °C and a spindle rotation rate (SC4-27) of 0.1 rpm.

## 3. Results

To address the research problem, it is first necessary to select a soluble zinc-containing component. This was accomplished by testing a number of relevant compounds (see Table 2)

The experimental data provided in Table 1 are generally in agreement with the reference data [43]. The highest solubility in phosphoric acid was demonstrated by metallic zinc and zinc sulfate heptahydrate. However, a cost-based comparison of these substances of equal purity showed that the latter is 65% less expensive [44,45]. For this reason, zinc sulfate heptahydrate was selected for this research.

**Figure 1:**
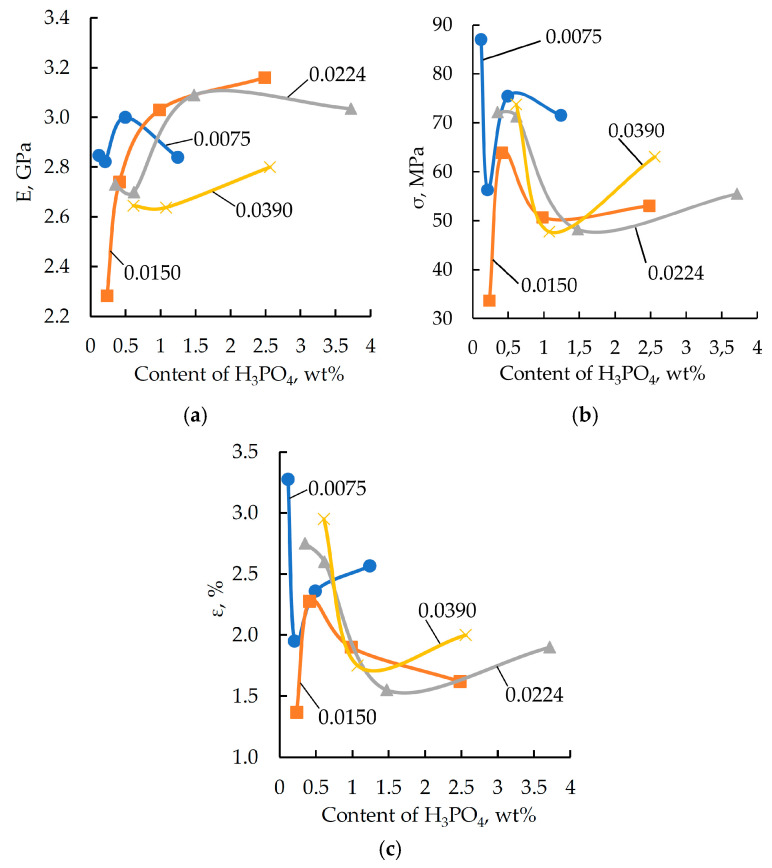
Elastic modulus (E) (**a**), breaking stress (σ) (**b**), and relative strain (ε) (**c**) obtained from a three-point bending test as a function of the orthophosphoric acid content in the samples. The DGEBA/TETA ratio was 100:10. The labels on the curves correspond to the average content (wt%) of H_3_PO_4_ in the modifier.

**Figure 2:**
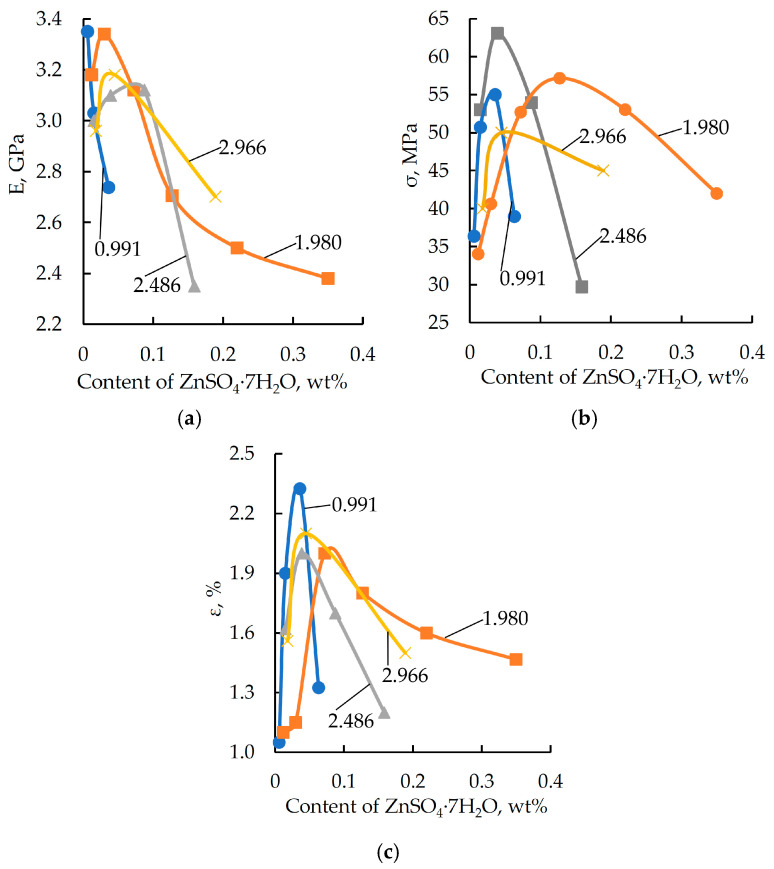
Elastic modulus (E) (**a**), breaking stress (σ_f_B__) (**b**), and relative strain (ε) (**c**) obtained from three-point bending tests as a function of zinc sulfate in the samples. The DGEBA/TETA ratio was 100:10. The labels on the curves correspond to the content (wt%) of ZnSO_4_·7H_2_O dissolved in 1.98 wt% H_3_PO_4_.

To optimize the binder formulation with respect to both the content of zinc salt in the modifier and the modifier itself, the components were analyzed for their impact on the stress–strain properties of the epoxy polymers. The results of the analysis are shown in Figure 1 and Figure 2.

As shown by the characteristic curves (see Figure 1 and Figure 2), the target physical and mechanical properties tend to improve with an increase in the concentration of both orthophosphoric acid and zinc salt in the formulation. However, once the modifying additive reaches a specific concentration, the polymer properties begin to deteriorate. A similar dependence was reported by the authors of [46,47] in their study on the effect of two-dimensional graphene nanoparticles on the mechanical properties of an epoxy composite. The strength enhancement was attributed to the ability of the solid particles to engage in interfacial interaction, leading to the formation of robust boundary layers. Additionally, the fracture surface area increases due to a change in the propagation path of the primary crack. The strength reduction is associated with the increased proportion of the particle–matrix interphase layer. Similar processes are presumably also taking place in the materials under investigation.

Formulations containing 0.991, 2.486, and 2.966 wt% of phosphoric acid and varying amounts of zinc sulfate were excluded from further studies, as these compositions do not provide the required level of elastic-strength properties. The maximum values of elastic modulus and breaking stress are characteristic of formulations containing 1.98 wt% of orthophosphoric acid and various amounts of zinc sulfate. Based on this result, formulations with the codes “0” and “21”–“25” were selected for further investigation (see Table 1).

**Figure 3:**
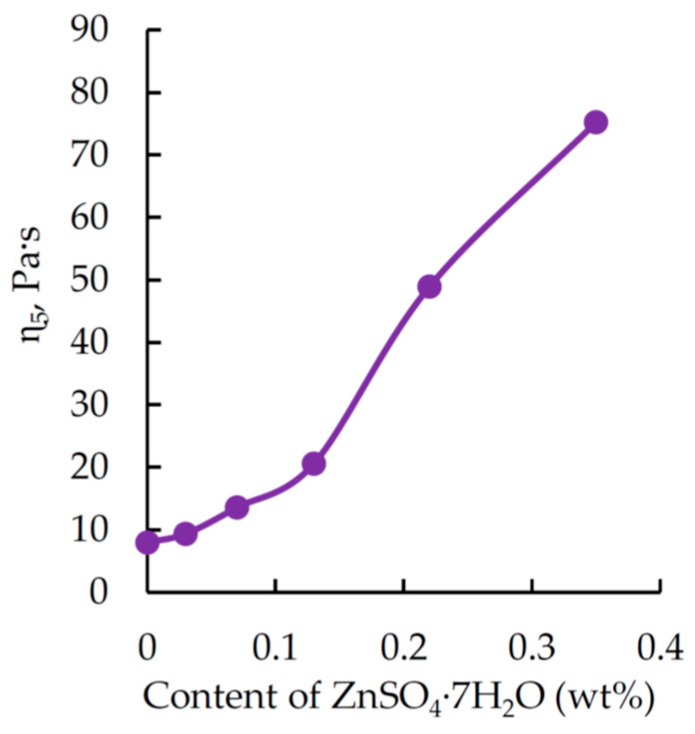
Dynamic viscosity 5 min after mixing the binder components as a function of ZnSO_4_·7H_2_O content (wt%) in the formulation. H_3_PO_4_ content (wt%): 1.98. DGEBA/TETA ratio: 100:10 (*wt*/*wt*).

The assessment of the technological viability of a binder is a prerequisite for selecting an optimal method of manufacturing composite materials with reactive matrices. For this assessment, the developed binders were subjected to a viscometric study designed to measure viscosity during the curing process. The compositions used for the study were prepared in accordance with the formulations in Table 2. The variation in dynamic viscosity of the binder as a function of ZnSO_4_·7H_2_O content in the formulation is shown in Figure 3.

Analysis of the data in Figure 3 shows that a phosphoric acid solution of zinc sulfate significantly increases the curing rate, causing the dynamic viscosity to reach a value of 75 Pa∙s within just 5 min. This markedly exceeds the optimal viscosity level for binders used for manufacture composites via vacuum methods [48,49]. This explains the need for adjustments to reduce viscosity; however, such adjustments would most probably require modifying the formulation, which is outside the scope of this research.

**Figure 4:**
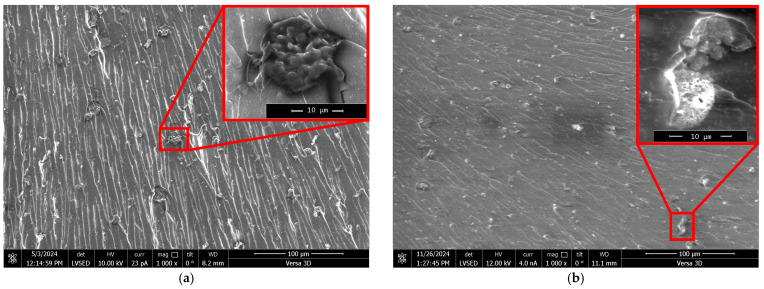

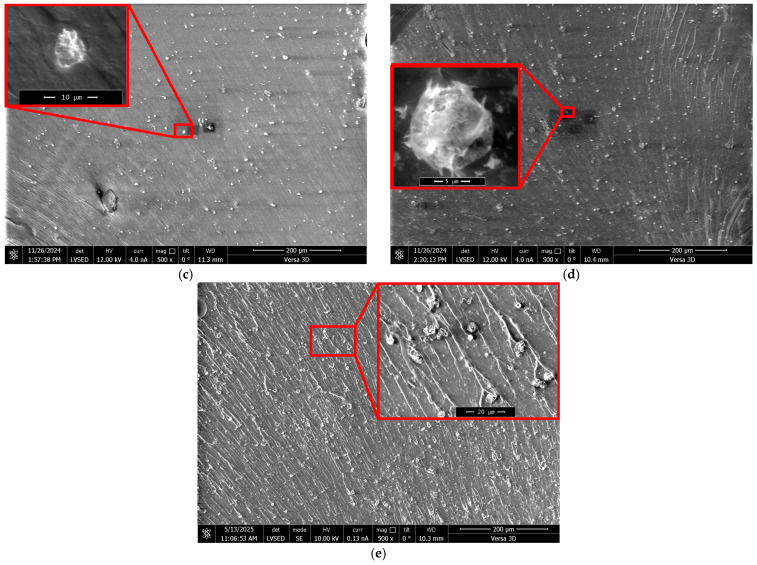
SEM micrographs of direct fracture surfaces of epoxy polymers (DGEBA:TETA ratio = 100:10) containing different amounts of ZnSO_4_·7H_2_O (wt%) dissolved in 1.98 wt% H_3_PO_4_: (**a**) 0.03, (**b**) 0.07, (**c**) 0.13, (**d**) 0.22, (**e**) 0.35. Regions with microparticles are outlined in red.

For a more in-depth study of the epoxy polymers structure, a fractographic analysis was conducted via scanning electron microscopy (SEM) on direct polymer fractures surfaces. The analysis results are shown in Figure 4.

Figure 4 shows that particles are present across the entire fracture surface. These particles are fairly uniformly distributed, without pronounced accumulation areas. It is evident that they formed in situ during the curing of the binder as a result of several successive and parallel chemical interactions of the modifier with DGEBA and TETA.

It should be noted that the fractographic analysis of direct polymer fractures based on SEM did not reveal any microdefects, such as pores or microchannels, which could be formed by the water present in the modifier. Its content in the modifier is 27.6 ± 0.5 wt%, which accounts for about 0.5 wt% of the entire epoxy composition. This is comparable to the moisture content in amine hardeners [50] and is unlikely to cause a substantial decrease in performance.

**Figure 5:**
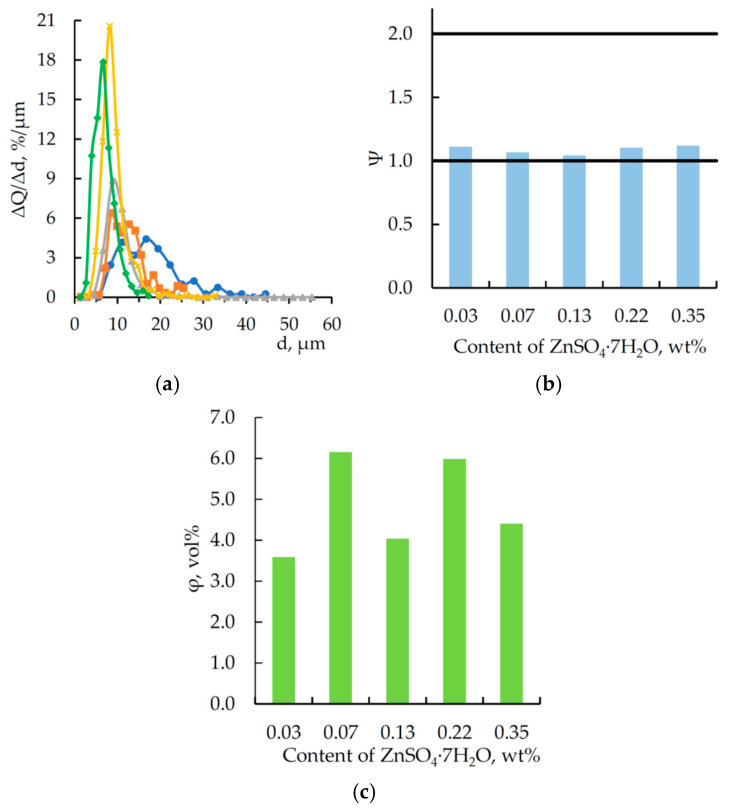
(**a**) Differential curves of the quantitative particle size distribution in polymer fractures (DGEBA/TETA—100:10) based on SEM data; calculated form factors (**b**), and (**c**) particles volume fraction as a function of ZnSO_4_·7H_2_O content in H_3_PO_4_ (1.98 wt%), wt%: 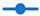
—0.03, 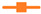
—0.07, 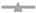
—0.13, 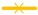
—0.22, 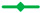
—0.35.

For particles observed in Figure 4, the key particle-size distribution metrics were analyzed, as shown by the data in Figure 5.

Analysis of the data in Figure 5a shows that with an increase in the content of zinc sulfate heptahydrate dissolved in orthophosphoric acid, the peak of the particle size distribution increases while the width of the differential curve decreases. This indicates a reduction in polydispersity and a decrease in the average particle size, alongside an increase in the number of particles. The likely explanation is that as the resin and hardener react with the modifier, the phosphoric acid content decreases, leading to the precipitation of a salt incompatible with the epoxy matrix. This salt acts as a nucleation site for microparticles during the curing process. Presumably, a higher content of zinc sulfate heptahydrate in the modifier accelerates the curing rate of the epoxy matrix, and particles agglomeration is limited because its rate is lower than that of the modifier’s chemical interaction with the epoxy matrix components.

According to the calculated data (see Figure 5b), the shape of particles in all samples was relatively close to spherical (Ψ → 1). Therefore, the volume fraction of microparticles in the material was calculated. The value of this parameter falls within an approximate range of 3.6–6.2 vol% and does not indicate a direct or inverse dependence on the content of zinc sulfate heptahydrate dissolved in orthophosphoric acid. Apparently, the modifier used in the experiment participates not only in the formation of microparticles but also in the curing of DGEBA. Thus, these processes are subject to a certain “competition”, which in turn is reflected in the particle size distribution.

The results of the energy-dispersive analysis of the polymer matrix and the particles contained within it are provided in Table 3 for a sample of one of the studied formulations.

Analysis of the data in Table 3 reveals clear differences in the elemental compositions of the polymer matrix and the particles. This is particularly apparent in the phosphorus content (23.7 times higher than in the matrix) and carbon content (9.3% lower than in the matrix), and may be due to the fact that particles were formed from the reaction products of phosphoric acid with the binder components. Notably, similar results were obtained from the analysis of all studied formulations.

**Figure 6:**
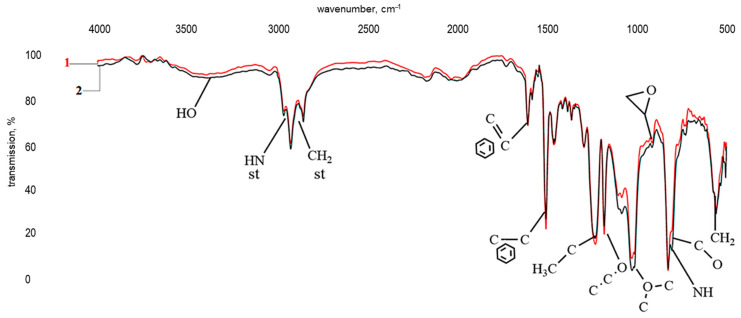
Infrared spectra of a bisphenol A diglycidyl ether sample cured with TETA (1) and a similar sample modified with a ZnSO_4_·7H_2_O solution (0.13 wt%) in H_3_PO_4_ (1.98 wt%) (2).

Figure 6 shows the results of infrared spectroscopy of a polymer prepared according to formulation 24 (see Table 1). Preliminary analysis indicated that the infrared spectra of samples differing only in the concentration of the modifying additive are similar.

The infrared spectrum of a polymer with formulation 24 (Figure 6, curve 2) is comparable to the spectrum of bisphenol A diglycidyl ether cured with triethylenetetramine (Figure 6, curve 1). It is characterized by absorption bands in the ranges 3269–3200 cm^−1^ and 765–800 cm^−1^, corresponding to stretching and bending vibrations of primary and secondary amine groups (-NH-), respectively. Wave numbers in the areas ranges 1608–1502 cm^−1^, 1228–1178 cm^−1^, and 1008 cm^−1^ correspond to stretching vibrations of the aromatic ring, the -C-O-C group of simple ethers, and methylene groups, and the band at 914 cm^−1^ is attributed to stretching vibrations of a -C-O oxirane group. Given the low intensity of the latter, the curing of the modified DGEBA can be said to be as complete as in reference sample “1”. This is further evidenced by a broad absorption band corresponding to vibrations of an OH group. No significant differences were observed between spectra “1” and “2”.

In view of this, to identify the specifics of chemical interaction leading to the in situ formation of microparticles, it would be expedient to combine binder components with the modifier according to the following patterns in proportions corresponding to the developed formulations (see Table 2):

DGEBA + (ZnSO_4_·7H_2_O solution in H_3_PO_4_) (1)

TETA + (ZnSO_4_·7H_2_O solution in H_3_PO_4_) (2)

**Figure 7:**
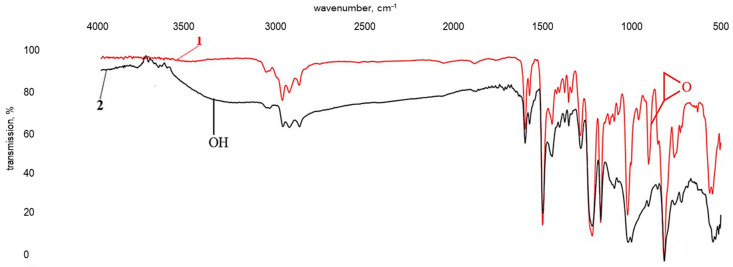
Infrared spectra of uncured DGEBA (1) and DGEBA modified with a ZnSO_4_·7H_2_O solution (0.13 wt%) in H_3_PO_4_ (1.98 wt%) (2).

**Figure 8:**
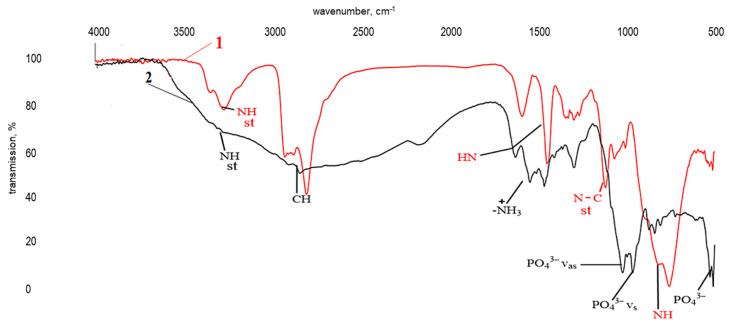
Infrared spectra of triethylenetetramine (TETA) (1) and TETA with ZnSO_4_·7H_2_O (0.13 wt%) in H_3_PO_4_ (1.98 wt%) (2).

The formation of a gel in the reaction (1) indicates an increase in molecular mass. The reaction following the pattern (2) proceeds very rapidly, even when the component is gradually added to triethylenetetramine. These specifics explain the need to conduct the synthesis step using an inert solvent, which in this case was toluene. After mixing the components, the reaction mixture was allowed to stand for at least 24 h, then filtered, washed and air-dried. The infrared spectra of the resulting products are shown in Figure 7 and Figure 8.

The infrared spectrum of modified bisphenol A diglycidyl ether (Figure 7, spectrum 2) comprises all absorption bands characteristic of epoxy resins, including those discussed above for spectrum 1 in Figure 6. However, the band intensity at 914 cm^−1^ is dramatically reduced, which indicates that the majority of epoxy groups participated in the reaction with phosphoric acid. This is also evidenced by the emergence of a broad, intensive band at about 3400 cm^−1^, corresponding to O–H stretching vibrations, and an absorption band at 1010 cm^−1^, characteristic of stretching vibrations of a P–O–C bond.

This allows us to suggest that the interaction of orthophosphoric acid with bisphenol A diglycidyl ether follows pattern (3):
(3)
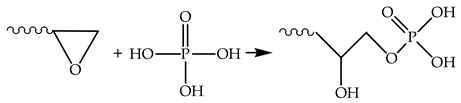



On the other hand, the possibility of etherification between phosphoric acid and a hydroxyl group in the epoxy resin should not be excluded. However, a higher intensity of the absorption band at about 3400 cm^−1^ points to an increase in the number of hydroxyl groups, rather than their depletion. The high reactivity of the oxirane ring and phosphoric acid suggests that the interaction may occur with one, two, or all three hydroxyl groups of the acid, which in turn is determined by the ratio of reagents and the reaction time. The presence of a weak absorption band at 914 cm^−1^ in spectrum 2 (Figure 7) indicates residual epoxy groups. In the composite structure, these groups presumably form chemical bonds with the polymer matrix. The matrix is apparently a product of the reaction of DGEBA, TETA, and a certain residual quantity of orthophosphoric acid. The presence of nitrogen detected during the SEM study of direct fractures of epoxy polymers confirms that the formation of microparticles involves not only epoxy resin but also triethylenetetramine.

Additionally, the ratio of epoxy to hydroxyl groups is approximately 9.4:1, indicating an excess of epoxy groups in the composition. This is the most likely reason why the particle shells are formed mainly by cross-linked products of the interaction between orthophosphoric acid and epoxy resin. To assess the ability of TETA to react with the modifying additive, the product obtained by mixing the components according to pattern (2) was subjected to infrared spectroscopy analysis (Figure 8).

Spectrum “2” in Figure 8 lacks pronounced absorption bands in the ranges 3269–3200 cm^−1^ and 765 cm^−1^, corresponding to stretching and bending vibrations of amine groups (-NH-) in secondary amines, respectively. Instead, a very broad absorption band is observed in the approximate range of 3600 to 1800 cm^−1^ characteristic of ammonium salts, including alkyl-substituted ones. There are also a number of absorption bands around 1632 cm^−1^, normally correlating with bending vibrations of ammonium cations (N^+^-H). The bands at 1027, 921, and 518 cm^−1^ are characteristic of the asymmetric (ν_as_), symmetric (ν_s_) stretching vibrations and asymmetric (δ_as_) bending vibrations of a $PO_{4}^{3-}$ group in trisubstituted orthophosphates. The data obtained confirm the ability of the modifying additive to react with the hardener, forming ammonium salts [51,52]:
(4)
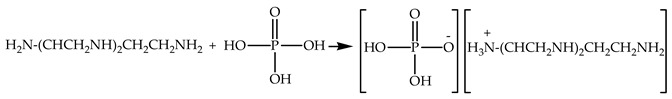



**Figure 9:**
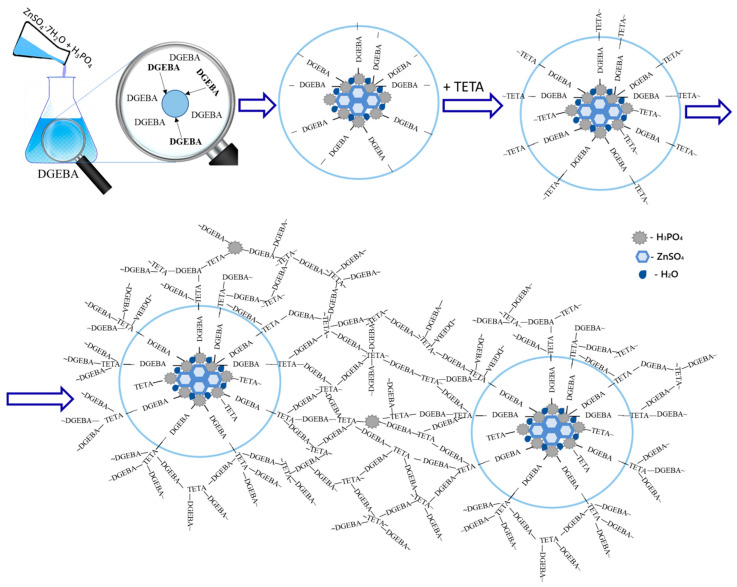
Scheme illustrating the interaction of an H_3_PO_4_ solution of ZnSO_4_·7H_2_O with DGEBA and TETA, and the structure of the resulting materials.

Based on the SEM and IR spectroscopy data, the following mechanism can be proposed to describe the interaction of DGEBA, TETA, and a solution of zinc sulfate heptahydrate in orthophosphoric acid (see Figure 9).

When oxirane cycles of the epoxy resin react with orthophosphoric acid, the acid concentration in the modifier decreases, leading to the formation of a salt that is incompatible with the epoxy matrix and subsequently acts as a crystallization site. The surface of these centers accumulates the products of the reaction between orthophosphoric acid and the epoxy resin. Consequently, some of the reactive groups remain unreacted due to steric hindrance and the decreased chemical activity of mono- and disubstituted orthophosphoric acid. Therefore, particle growth also involves triethylenetetramine, which, in turn, forms chemical bonds between the particles and the polymer matrix. The latter is most likely a product of the reaction between DGEBA, TETA, and a certain residual amount of orthophosphoric acid.

The gel fraction content of approximately 98.5 ± 0.4 wt% remains roughly equal for both the reference and modified samples. This result is largely expected, as the structure of the polymer matrix (outside the particles) forms primarily through the interaction between DGEBA and TETA. At the same time, these results support the previously made suggestion that the particles are chemically bonded to the matrix.

**Figure 10:**
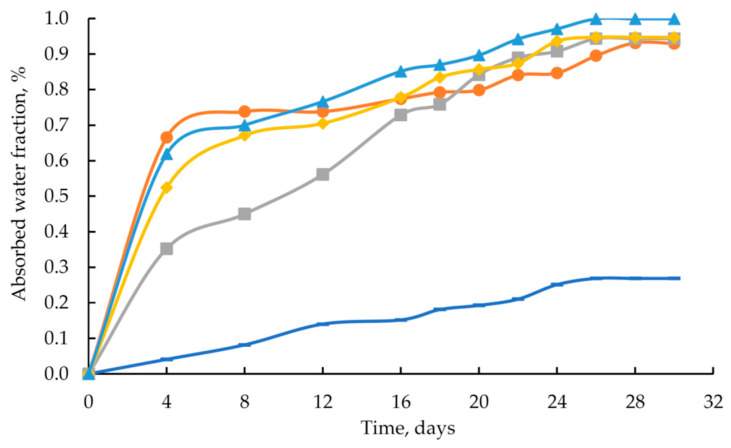
Absorbed water content as a function of exposure time for epoxy polymers (DGEBA/TETA = 100:10): (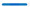) unmodified reference sample; (B–F) samples modified with H_3_PO_4_ (1.98 wt%) containing different amounts of ZnSO_4_·7H_2_O: (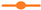) 0.03 wt%, (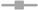) 0.07 wt%, (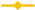) 0.13 wt%, (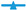) 0.22 wt%.

Since orthophosphoric acid, being the key component of the modifying additive, is fully miscible with water, it is practical to evaluate the water absorption of the developed polymers (see Figure 10).

The data in Figure 10 indicate that the modified samples exhibit lower resistance to an aqueous environment than the reference sample. At the same time, the maximum absorbed water content for polymers containing an additive based on orthophosphoric acid and zinc salt is 1.00%.

To evaluate the potential for acid leaching from the material, the pH of the aqueous medium obtained after immersion of the exposure of the cured epoxy polymer samples was measured (see Table 4).

The observed alkaline shift in pH indicates that the concentration of hydrogen ions (H^+^) in the solution does not increase. This can be attributed to the leaching of unreacted triethylenetetramine. At the same time, the unreacted hardener promotes an increase in the sorption capacity of the polymer samples towards water. This explains the higher water absorption of the modified samples (see Figure 10).

XRF analysis of the medium also confirmed that no leaching of the modifier leaching of the in water.

**Figure 11:**
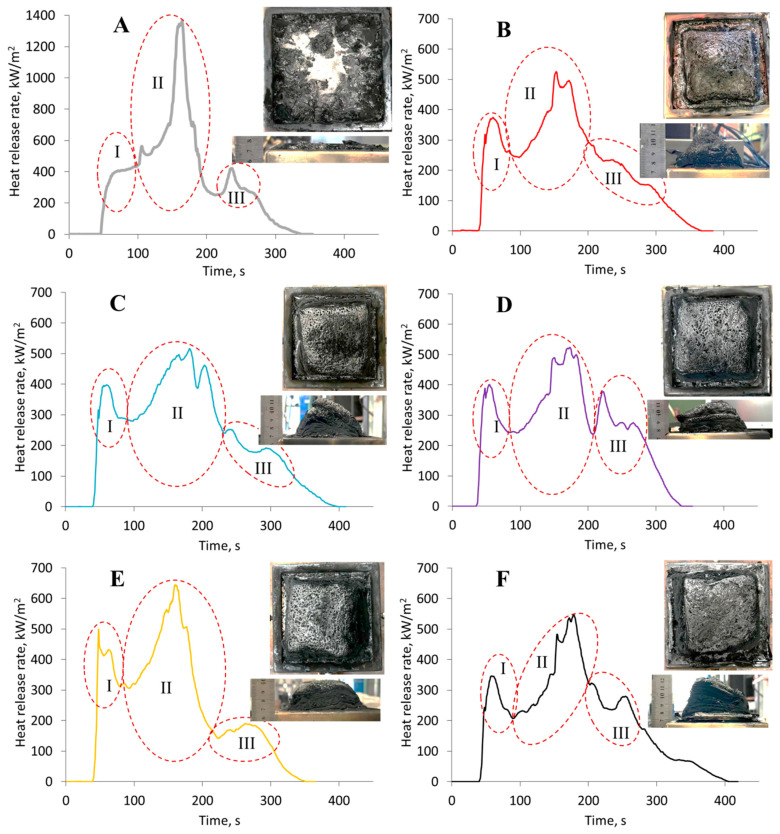
Heat release rate (HRR) as a function of time for epoxy polymer samples (DGEBA/TETA = 100:10) under a heat flux of 50 kW/m^2^: (**A**) unmodified reference sample; (**B**–**F**) samples modified with H_3_PO_4_ (1.98 wt%) containing different amounts of ZnSO_4_·7H_2_O: (**B**) 0.03 wt%, (**C**) 0.07 wt%, (**D**) 0.13 wt%, (**E**) 0.22 wt%, (**F**) 0.35 wt%.

The impact of microparticles on the flammability of the polymers was assessed using a cone calorimeter test. The results are provided in Figure 11 and Table 5.

For all curves shown in Figure 11, three areas with complex peaks can be identified. The first peak (area I) corresponds to the flame combustion of the material. The maximum of this peak coincides with the beginning of char layer formation on the surface of the samples. It is noteworthy that during cone calorimeter testing, materials modified with microparticles exhibit substantially greater swelling; for the reference sample, the char layer height does not exceed 10 mm, whereas modified samples occasionally form a char layer greater than 50 mm thick, with sufficient strength to prevent surface entrainment. The amount of char residue obtained from cone calorimeter testing of modified samples is approximately 1.7–2.2 times greater than that of unmodified samples. The increased thickness of this layer, which possesses heat-insulating and barrier properties, slows the heating of the underlying layers, thereby reducing the rate of decomposition and the intensity of heat release, while preventing the release of hot pyrolysis products. Evidence of this can be seen in the area of stationary heat release before the onset of the second complex peak, which is governed by a balance between the exothermic oxidation of condensed pyrolysis products and the endothermic decomposition of the material. According to [53,54,55,56], the temporal characteristics of this area depend on the quality and thermophysical properties of the char and the heat transfer conditions. It is clear that the curve reflecting the pyrolysis of unmodified samples (see Figure 11A) effectively lacks such an area due to the prevalence of exothermic processes.

The second complex peak (area II) corresponds to flameless heterogeneous combustion after flame extinction—smoldering. During the pyrolysis of the unmodified material (see Figure 11A), it is evident that this process is associated with a significantly higher heat release rate compared to samples containing solutions of zinc sulfate heptahydrate in phosphoric acid. This is likely a consequence of two factors. First, brittle fracture of the char layer and its surface entrainment increase the influx of atmospheric oxygen into the pyrolysis zone, thereby intensifying oxidation. Second, the reduced intensity of heat release for the modified samples is associated with the presence of phosphorus-containing fragments and structures, especially the microparticles discussed above.

The emergence of a complex peak in area III (see Figure 11) is atypical for cone calorimeter test results [53]. Presumably, after the cessation of flameless combustion and some cooling of the char layer, further cracking occurs, allowing atmospheric oxygen to access the char in areas near the instrumentation. These areas become further oxidized, leading to the emergence of exothermic peaks.

It should be noted that the modification under study generally increases the char residue and also markedly reduces thermal effects during combustion. This is evident, in particular, from the reduction in the total heat release by 39.5%, the mean heat release rate by 39.6%, and the peak heat release rate by 59.8%. A notable reduction was observed in the fire growth rate (FIGRA)—by a factor of 2.4–3—and in the effective heat of combustion—by 28.5%, which indicates a decrease in the fire hazard of the modified materials. Additionally, there was a decrease in the total smoke release by 30%.

Such fire hazard indicators as total heat release (THR, MJ/m^2^) and fire growth rate (FIGRA) are the primary characteristics determining the intensity and dynamics of fire development when these materials constitute the main fire load [54]. This approach is used by fire safety engineers worldwide for both fire hazard assessment of materials and computational fire modeling of real-life facilities [54]. These parameters have been successfully incorporated into relevant European Union regulations. While the obtained data do not allow for specific conclusions regarding the fire hazard classification of these materials according to the European standard [57], the general decrease in key metrics—such as heat release rate, fire propagation dynamics, and smoke production—by 30–70% demonstrates a considerable reduction in the fire hazard of the modified materials compared to the feedstock.

Nevertheless, no obvious correlation was found between the above-mentioned fire test results and the particle size distribution of microparticles formed during curing. No correlation with the volume fraction of the particles was found either. In other words, the processes leading to heat release and/or absorption during the combustion of the studied samples depend not on the size, distribution, or quantity of phosphorus-containing particles, but on the total phosphorus content in the material’s structure. This finding is generally consistent with the literature data, specifically [8,9,10]. Furthermore, changes in the granulometric composition of the microparticles formed during curing were reflected in higher numerical values of the limiting oxygen index, which is consistent with studies [58].

**Figure 12:**
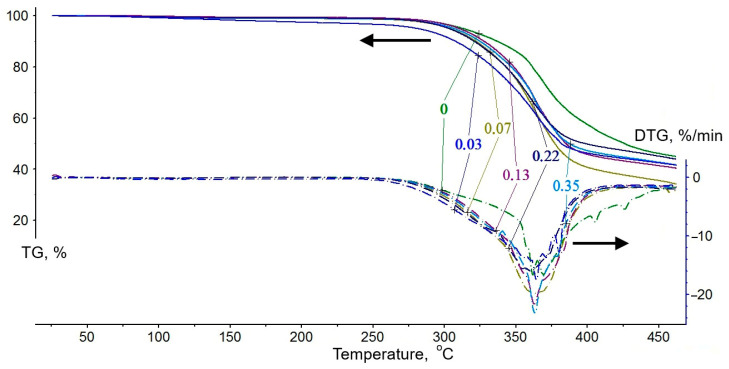
Results of thermal gravimetric analysis (TGA) of polymer samples (DGEBA/TETA—100:10) modified with a phosphoric acid solution of zinc sulfate heptahydrate. The curve designations correspond to the content of ZnSO_4_·7H_2_O (wt%) in H_3_PO_4_ (1.98 wt%).

The results of thermogravimetric analysis (TGA), reflecting the thermal-oxidative degradation at the first stage—which characterizes the onset of material decomposition and the fire-retardant mechanism—are presented in Figure 12 and Table 6.

Analysis of the curves in Figure 12 leads to the conclusion that the rate of thermal oxidation increases for all samples in the temperature range of 276–401 °C, as evidenced by a significant mass loss of 40.4 to 51.5%. According to the data in Table 5, the sample without the modifier is characterized by the highest initial process temperature ($T_{\mathrm{st}}^{I}$)—294 °C—and the lowest mass loss in the first stage—40.4%. The introduction of a phosphoric acid solution of zinc sulfate heptahydrate into the polymer formulation shifts the onset of thermal oxidation to a lower temperature range, with $T_{\mathrm{st}}^{I}$ values between 276 and 289 °C. Consequently, there is a notable increase in mass loss in the samples (44.3–51.5%) due to first-stage degradation. At the same time, the temperatures at which the peak mass loss rate (T_m_) is reached are relatively close—approximately 362 ± 2 °C. Notably, no correlation is observed between the zinc sulfate content in the polymer-modifying solutions and the temperatures characterizing the thermal-oxidative degradation process. Therefore, it is likely that a decrease in these temperatures is primarily associated with the presence of alkyl-substituted ammonium salts formed during the interaction of triethylenetetramine and orthophosphoric acid. As is known, thermal oxidation of such compounds occurs at lower temperatures than the degradation of polymers based on epoxy matrices [17,59]. At the same time, a certain increase in the peak mass loss rate (PMLR) is observed with a rise in the zinc sulfate content. This indicates an acceleration of thermal-oxidative degradation reactions, leading to an increased yield of a protective char layer during the first stage of degradation. During testing of samples based on DGEBA and TETA, this stage is completed at a temperature of 401 °C, whereas the presence of the modifier reducing it to 382 °C. It should be noted that these values are significantly lower than the self-ignition temperature of epoxy polymers, which is 470 °C according to reference data [60]. Most probably, the fire-retardant action of the modifier consists of the earlier and faster formation of a protective char layer in the first stage of thermal-oxidative degradation, with an increasing yield. This, in turn, leads to the formation of a more voluminous and thermally stable foam char layer, as confirmed by the cone calorimeter tests. These effects contribute to a reduction in the flammability and fire hazard of polymer materials [61].

## 4. Conclusions

A novel method for modifying epoxy polymers was developed using a modifier comprising a solution of zinc sulfate heptahydrate in phosphoric acid. Its introduction into DGEBA and subsequent curing with TETA facilitated the in situ synthesis of microparticles (6–16 µm), a phenomenon not previously reported. Increasing the zinc salt content reduced particle polydispersity and average size, while increasing their number; the particle morphology remained near-spherical.

A mechanism is proposed wherein the interaction of epoxy oxirane cycles with phosphoric acid lowers its effective concentration, precipitating the incompatible zinc salt as crystallization centers. Reaction products accumulate on their surfaces, with some phosphate groups remaining unreacted due to steric hindrance. TETA then integrates the particles into the network by forming bonds with the matrix—a product of DGEBA, TETA, and residual acid.

Increasing the modifier concentration raised the limiting oxygen index (up to 23.9 vol%) and the peak mass loss rate during thermo-oxidative degradation, due to earlier char formation acting as a barrier. The overall phosphorus content was found to influence combustion-related thermal processes more than the specific particle size or distribution.

In summary, optimizing the modifier composition significantly reduces flammability while preserving satisfactory physico-mechanical properties. The developed formulations show promising potential as matrix binders for fiber-reinforced composites based on glass or carbon fabrics.

## Figures and Tables

**Table 1:** Formulations of the developed epoxy polymers.

Formulation Number	DGEBA, wt%	TETA, wt%	Modifying Additive, wt%	
			ZnSO_4_·7H_2_O	H_3_PO_4_
0	90.90	9.10	-	-
1	89.77	8.98	0.0075	1.2425
2	90.45	9.05		0.4925
3	90.71	9.07		0.2075
4	90.80	9.08		0.1175
5	88.64	8.86	0.0150	2.4850
6	90.00	9.00		0.9850
7	90.52	9.05		0.4130
8	90.68	9.07		0.2350
9	87.51	8.75	0.0224	3.7176
10	89.55	8.95		1.4775
11	90.33	9.03		0.6176
12	90.57	9.06		0.3516
13	88.64	8.86	0.0390	2.11
14	88.55	8.85		2.5610
15	89.90	8.99		1.0760
16	90.32	9.03		0.6110
17	90.00	9.00	0.006	0.994
18	90.00	9.00	0.015	0.985
19	89.97	9.00	0.036	0.994
20	89.95	9.00	0.058	0.992
21	89.09	8.90	0.03	1.98
22	89.05	8.90	0.07	1.98
23	88.99	8.90	0.13	1.98
24	88.95	8.85	0.22	1.98
25	88.84	8.83	0.35	1.98
26	88.64	8.86	0.015	2.485
27	88.55	8.85	0.039	2.561
28	88.64	8.86	0.087	2.413
29	88.50	8.85	0.159	2.491
30	88.18	8.82	0.018	2.982
31	88.18	8.82	0.045	2.955
32	88.05	8.80	0.189	2.961

**Table 2:** Assessment of acid solubility of zinc-containing components.

Component A	Component B	Compatibility Assessment
Orthophosphoric acid (H_3_PO_4_)	Metallic zinc (Zn)	Dissolves (5 wt% or more) at 20–25 °C within 24 h. Releases gas during dissolution.
	Zinc oxide (ZnO)	Dissolves (up to 2 wt%) only after 12 h’ exposure to 80 °C, but forms a scale deposit on the vessel walls 72 h after dissolution.
	Zinc phosphate (Zn_3_(PO_4_)_2_)	Dissolves (up to 2 wt%) only after 12 h’ exposure to 80 °C, but precipitates within 7 days as a white sediment apparent due to opalescence.
	Zinc metasilicate (ZnSiO_3_)	Insoluble (both at room temperature and upon heating)
	Zinc sulfate 7-hydrate (ZnSO_4_·7H_2_O)	Dissolves (up to 20 wt%) at 20–25 °C within 24 h. Remains stable during storage.

**Table 3:** Results of energy-dispersive analysis of a direct fracture of an epoxy polymer (DGEBA/TETA = 100:10) modified with a ZnSO_4_·7H_2_O solution (0.22 wt%) in H_3_PO_4_ (1.98 wt%).

Element	Theoretical Content	Average Element Content, wt%	
		Polymer Matrix	Particle
C	72.8	82.0	73.9
O	17.8	14.1	14.3
N	3.39	3.6	4.0
P	0.46	0.3	7.1
S	0.03	0	0.3
Zn	0.06	0	0.4
H ^1^	5.46	-	-

^1^ Not recorded in the energy-dispersive analysis setting.

**Table 4:** Results of pH measurement of water after exposition.

Medium/Formulation	Distilled Water	0	1	2	3	4
pH	6.02	6.50	6.60	6.80	7.00	7.40

**Table 5:** Results of fire testing of the developed epoxy polymers.

Content of ZnSO_4_· 7H_2_O, wt%	Content (Theor.) of P, wt%	Content (Theor.) of Zn, 10^3^ wt%	LOI, vol%	THR, MJ/m^2^	meanHRR, kW/m^2^	PHRR, kW/m^2^	FIGRA, kJ/m^2^c^2^	Q_ef.,_ kJ/g	TSR m^2^/m^2^	Char Residue from Cone Calorimeter Test, wt%
-	-	-	19.0	117.4 ± 1.3	361.3 ± 14.5	1254.6 ± 107.6	8.5 ± 0.2	21.4 ± 1.4	2242 ± 91	8.7 ± 0.5
0.03	0.46	6.8	23.1	81.9 ± 14.8	168.0 ± 14.0	517.5 ± 8.0	3.1 ± 0.3	16.7 ± 2.4	1920 ± 65	14.9 ± 0.2
0.07	0.46	15.9	23.2	86.3 ± 5.3	174.5 ± 8.5	512.4 ± 2.9	2.9 ± 0.1	17.9 ± 0.2	1849 ± 113	18.7 ± 2.1
0.13	0.46	29.6	23.3	77.7 ± 6.5	172.5 ± 1.5	534.7 ± 11.1	3.1 ± 0.1	16.6 ± 1.9	1570 ± 164	19.5 ± 0.2
0.22	0.46	50.0	23.5	73.0 ± 1.4	174.0 ± 13.0	610.9 ± 33.9	3.5 ± 0.5	15.3 ± 0.1	1610 ± 193	15.1 ± 0.5
0.35	0.46	79.6	23.9	71.1 ± 9.1	183.5 ± 3.5	504.0 ± 42.6	2.8 ± 0.3	15.3 ± 1.7	1575 ± 155	18.9 ± 3.2

DGEBA/TETA ratio (*wt*/*wt*)—100:10. H_3_PO_4_ content—1.98 wt%. Applied heat flux during cone calorimeter testing—50 kW/m^2^; LOI—limiting oxygen index; THR—total heat release; meanHRR—mean heat release rate; PHRR—peak heat release rate; TSR—total smoke release; FIGRA—fire growth rate [53,54,55,56]; Q_ef._—effective heat of combustion [56].

**Table 6:** Key parameters from the thermogravimetric analysis of the developed epoxy polymers.

Content of ZnSO_4_·7H_2_O, wt%	Content (Theor.) of P, wt%	Content (Theor.) of Zn, 10^3^ wt%	$T_{\mathrm{st}}^{I}$, °C	$T_{\mathrm{fin}}^{I}$, °C	PMLR, %/min	T_m_, °C	Δm^I^, %
-	-	-	294	401	20.6	360	40.4
0.03	0.46	6.8	276	384	17.5	364	46.5
0.07	0.46	15.9	295	385	19.8	364	51.5
0.13	0.46	29.6	298	385	21.6	363	47.3
0.22	0.46	50.0	281	382	16.0	361	44.3
0.35	0.46	79.6	289	382	23.1	363	45.4

DGEBA/TETA ratio (*wt*/*wt*)—100:10. H_3_PO_4_ content—1.98 wt%; $T_{\mathrm{st}}^{I}$—initial first-stage temperature of thermal-oxidative degradation marking the beginning of mass loss intensity on the DTG curve; $T_{\mathrm{fin}}^{I}$—final first-stage temperature of thermal-oxidative degradation based on the point of intersection of tangents drawn through the TG curve bends; PMLR—peak mass loss rate; T_m_—PMLR temperature; Δm^I^—mass loss after the first stage of thermal-oxidative degradation.

## Data Availability

The original contributions presented in this study are included in the article. Further inquiries can be directed to the corresponding author.

## Abbreviations

The following abbreviations are used in this manuscript:

PMC	polymer matrix composites
DGEBA	bisphenol A diglycidyl ether
TETA	triethylenetetramine
LOI	limiting oxygen index
SEM	scanning electron microscopy
THR	total heat release
meanHRR	mean heat release rate
PHRR	peak heat release rate
TSR	total smoke release
FIGRA	fire growth rate
Q_ef._	effective heat of combustion
$T_{\mathrm{st}}^{I}$	initial first-stage temperature of thermal-oxidative degradation marking the beginning of mass loss intensity on the DTG curve
$T_{\mathrm{fin}}^{I}$	final first-stage temperature of thermal-oxidative degradation based on the point of intersection of tangents drawn through the TG curve bends
PMLR	peak mass loss rate
T_m_	PMLR temperature
Δm^I^	mass loss after the first stage of thermal-oxidative degradation
